# Supporting Healthy Aging through the Scale-Up, Spread, and Sustainability of Assistive Technology Implementation: A Rapid Realist Review of Participatory Co-Design for Assistive Technology with Older Adults

**DOI:** 10.1177/23337214211023269

**Published:** 2021-06-11

**Authors:** Simon Carroll, Karen Kobayashi, Matilde N. Cervantes, Shannon Freeman, Manik Saini, Shannon Tracey

**Affiliations:** 1 University of Victoria, BC, Canada; 2 University of Northern British Columbia, Prince George, Canada; 3 Ministry of Health, Victoria, BC, Canada

**Keywords:** healthy aging, gerontology, assistive technology, rapid realist review, participatory co-design, implementation

## Abstract

**Objective:** To identify the key mechanisms, contexts, and outcomes that drive the successful participatory co-design of assistive technologies. **Method:** A rapid realist review was conducted using a systematic search strategy. After screening, a final set of 28 articles were included. Articles were analyzed for evidence relevant to our initial program theory (IPT), and context-mechanism-outcome configurations were developed, resulting in a revised program theory. **Results:** All 28 articles included were highly relevant to the IPT, and had sufficient detail regarding the process of participatory co-design. The findings of this review highlight several key context-mechanism-outcome configurations as potential patterns in the data under the two dimensions of the evolving program theory: *knowledge integration* and the *ethico-political* dimension. **Discussion:** This review revealed the key mechanisms of *mutual awareness, mutual learning, trust*, and *reciprocity* that need to be taken into account in AT development and assessment. We concluded that participatory co-design requires a restructuring of power relations between end-users and those traditionally in control of technology design. These findings inform the development and assessment of AT for older adults and help guide policy/decision-makers to move forward with the now urgent agenda for scale-up and spread, initiated by the burning platform of the COVID-19 pandemic.

## Introduction

The WHO has estimated that around one billion people require some form of assistive technology (AT), defined as “assistive products and related systems and services developed for people to maintain or improve functioning and thereby promote well-being” (WHO, 2020), and that this number is likely to double by 2050; it also estimates that about 90% of people deemed to require an AT, do not currently have access to them (WHO, 2020). Much of this current and future demand is driven by an aging population. A large part of the access problem has to do with financial means, with many people, especially in the developing world, unable to purchase ATs that are provided on the private marketplace; and, even in developed societies, many older adults find themselves unable to afford more expensive available ATs (de Witte et al., 2018). This is a major policy barrier, with many health systems unwilling to provide life-enhancing ATs on a sliding scale at the point of use, based on need. However, ATs are endemically susceptible to implementation failures that point to much more complicated issues than simply the lack of access based on affordability. Many failures have to do with the fact that ATs are: (1) not fit for purpose and so are either not adopted or are quickly abandoned by users; or (2) simply not able to be integrated with existing care systems (informal and formal); or (3) not situated in regulatory and procurement contexts that are favorable to their scale-up and spread (Greenhalgh, Fahy & Shaw, 2018). These barriers and challenges are generated by the fact that an AT implementation takes place within complex, multi-level social systems, where a variety of stakeholders (older adults, their informal carers, professional/formal carers, health and social system managers, researchers, technology developers, and high-level policy makers) must negotiate, collaboratively, the progress of an AT, from initial exploration and ideation, through all the phases of development and implementation. As an AT moves along this complex, non-linear implementation process, it becomes, itself, part of a socio-technical system, or assemblage, that has to be stabilized, in order to scale-up, spread, and sustain itself materially and socially, embedding itself in a pre-existing health and social care system (Greenhalgh, Jackson, et al., 2016; Greenhalgh, Shaw, et al., 2016).

The theoretical potential of AT to help overstretched health and social care systems and extend and enhance the quality of life of older adults, seems almost limitless, as the proliferation of technical solutions grows exponentially, covering multiple aspects of daily life (e.g., bodily function, mobility and transport, communication, caregiving) that are foundational to aging in place, independently (Mincolelli et al., 2019). However, multiple barriers to the equitable implementation of assistive technologies persist for older adults. Recent work in Canada has pointed to some key barriers to the equitable implementation of AT, with researchers advocating for public engagement and involvement in identifying needs, challenges, and solutions, for more equitable implementation (Mattison et al., 2020; Wang et al., 2020). A common theme from the findings of this work is the need for more participation of end-users, and collaboration across system levels between multiple stakeholders. As Murphy et al. (2017) highlight, these collaborations can lead to elicit requirements for the design of AT systems to develop effectively as integrated care solutions.

In this review, we argue that an emphasis on older adult engagement and participation in the policy process is necessary, yet we describe a more comprehensive perspective on the role of participation in AT implementation, using the multi-level, ecological approach of the Non-Adoption, Scale-up, Spread, and Sustainability (NASSS) framework (Greenhalgh et al., 2017; Greenhalgh et al., 2020). This framework identifies multiple, interrelated domains as part of a complex adaptive system, within which AT implementation (or other technology implementation initiatives) must occur. In this paper, we are using this framework as a stepping off point, as it orients us to the different perspectives that our set of stakeholders (as listed above) have, based on what level of this complex adaptive system they primarily act from. It also orients us to some structural features of the *context* of AT implementation (features of everyday residential life for older adults; work process contexts for formal caregivers; the value proposition for AT developers; organizational culture and strategic priorities for health and social services; and, the macro-level system pressures on politicians and high-level policymakers).

One potential strategy for strengthening implementation for ATs that is increasingly advocated for within the field of gerontechnology is to use participatory co-design processes (PCD) in the development of AT. Although there are several designations that are closely related to this approach (e.g., user-centered design, co-design, participatory design, co-creation, co-production), we are using PCD in this study as it captures the essential nature of the enterprise, which involves designing ATs, together (co-) with older adults and other stakeholders, using explicitly “participatory” processes. In fact, many criticisms of “user-centered” and “co-design” projects are aimed at their inadequate ethical and methodological commitments to genuine participation (Grigorovich et al., 2021; Merkel & Kucharsky, 2019; Peine & Neven, 2019; Sumner et al., 2020). The rationale for using PCD is based on the assumption that these processes increase older adult engagement with the technologies and enhance the design process by including iterative feedback from these end-users. It has also been suggested that a wider array of stakeholders (as outlined above) need to be involved in these PCD processes, so that the complex implementation challenges we have discussed can be addressed and incorporated within the design and implementation of AT. However, both the calls made for this strategy to be more widely employed, and the few empirical reviews of such attempts in the field of gerontechnology, have often suffered from a lack of specificity about just what is to count as a “participatory co-design” approach (Merkel & Kucharski, 2019). Thus, traditional systematic review approaches, which simply measure outcomes against the presence or absence of a PCD approach are inadequate (cf. Greenhalgh, Thorne & Malterud, 2018). As there is no universal agreement about what makes up a PCD effort, it is hard to know whether there has been any real fidelity to the approach in any one case example. Second, it is not always clear what is to count as the “outcome(s)” of PCD. Is it higher rates of “acceptance,” “usability,” “adoption,” “scale-up,” “spread,” or “sustainability”? Or some combination of all? Or some other set of relevant outcomes? Perhaps, it is simply the “engagement,” “empowerment,” or increase in “autonomy’ and “independence” of older adults? While there are some examples of theoretically attuned explications of PCD (Spinuzzi, 2005; Fischer et al., 2020), in practice, it is still the case that PCD is most often applied loosely as an “approach” that is largely unspecified in its methodological details. This led our research team, which was funded to carry out an implementation science project on ATs for older adults, to use the initial stage of our project to conduct a rapid review of the relevant literature, aimed at better understanding what the key mechanisms were that drove successful examples of participatory co-design processes in the development and implementation of ATs for older adults. We were also cognizant of the fact that these mechanisms may or may not be effective (i.e., “triggered”), depending on who was using them and under what circumstances or contexts. Finally, we were interested in the kind of outcomes participatory co-design was meant to produce. What was needed was a theoretically-driven review methodology, and we chose the rapid realist review (RRR) methodology outlined by Saul et al. (2013).

## Methods

We followed the suggested composition of an RRR team (Saul et al., 2013), including a project manager, local reference group, expert panel, librarian, review team, synthesis lead, and academic or research lead. The RRR was conducted using RAMESES guidelines (Wong et al., 2013) and the steps for realist reviews outlined in Pawson et al. (2005). A RRR is a useful tool to generate knowledge synthesis with transferable theoretical findings that can be applied in different contexts. Indeed, the program theories RRR’s develop include considerations of how changes in context may interact with mechanisms to produce outcomes of interest. Program theories help to better understand intended and unintended outcomes resulting from changes in context and their resultant interactions with mechanisms (Saul et al., 2013). Our initial program theory (IPT) was derived from several key sources including a seminal theoretical overview of participatory design (Spinuzzi, 2005), and two recent systematic reviews, one on participatory design in gerontechnology (Merkel & Kucharsky, 2019), and one on the more general topic of “co-creation” in community-based health services (Greenhalgh, Jackson, et al., 2016; Greenhalgh, Shaw, et al., 2016).

We conducted an initial scoping of the literature searching for empirical evidence relevant to our IPT. As a preliminary task of the overall project, we identified the role of “participatory,” “co-“ or “user-centered” design processes as a key strategy for AT, culminating in a focus on the topic of PCD of ATs with older adults to understand the underlying mechanisms that related to the IPT.

This rapid realistic review used the six steps aligned with the Saul et al. (2013) RRR procedure and the Pawson et al. (2005) realist review stages. The six steps consist in clarifying the scope, searching for evidence, quality appraisal, extracting the data, synthesizing the data, and disseminating the findings.

The articles were selected according to their relevance to the contextual factors and mechanisms related to our IPT. Articles needed to achieve two goals: (1) to test the initial program theory in terms of its two identified dimensions; (2) to identify key Context-Mechanism-Outcome-Configurations (CMOCs) related to those dimensions, or other unidentified dimensions of the program theory. The overarching Research Questions guiding our review are as follow,

1. What are the key mechanisms that drive successful co-design/co-production processes for effective assistive technology implementation and use among older adult populations?
2. What are the important contexts which determine whether the mechanism produces the intended outcome? (Modified from Papoutsi et al., 2020)

To identify the relevant literature for this review, three journals were used for hand searching: Ageing and Society, Computer Supported Cooperative Work (CSCW), and Gerontechnology. Also, electronic databases were searched: AgeLine, BSC, the Cumulative Index to Nursing and Allied Health Literature (CINAHL), MEDLINE^®^, PsychINFO, the Sociological Abstracts, and Web of Science. Citations were imported into COVIDENCE^®^ data management software to track the screening, full text review, and data extraction process. Duplicate studies were identified and removed.

**Figure 1.**
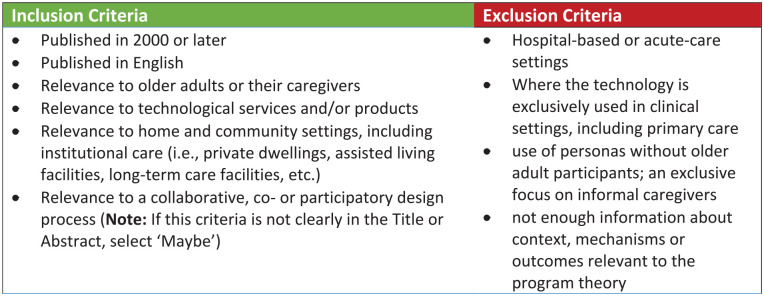
Screening criteria.

A multi-pronged, iterative search strategy was adopted for this review. A set of initial screening criteria was used to determine the potential relevance of documents based on the assessment of titles, keywords, and abstracts (see Figure 1 above). The documents were categorized as one of the following by the reviewer: Include—Article meets all the criteria; Maybe—It is not possible to determine or Exclude—Article does not meet one or more of the criteria. Documents identified as “maybe” were reviewed by the review team for consensus. The documents were screened and assigned a category using COVIDENCE^®^. All documents were screened by at least two reviewers to ensure consistency. Any inconsistencies were resolved through a discussion among the three members of the review team until consensus was reached. Once extraction was complete, the review team summarized the key themes and findings. At this stage, the reviewers focused on the “contextual factors and mechanisms that impact outcomes, and how context and mechanisms interact” (Saul et al., 2013). We created a matrix with contextual factors and mechanisms, then findings were grouped by similar contextual changes and “how they trigger mechanisms to produce outcomes” (Saul et al., 2013). The review team met twice weekly throughout this process to discuss the groupings to ensure validity and consistency of the findings. The local reference group was engaged simultaneously to ensure relevance.

**Figure 2.**
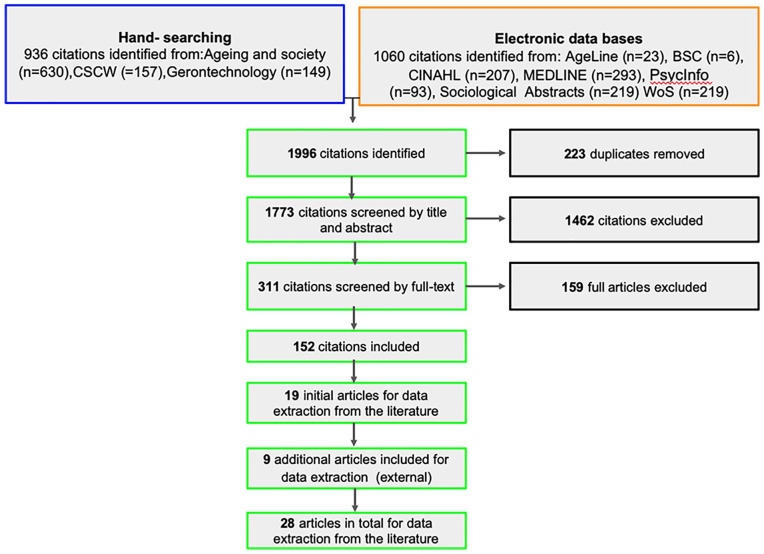
Flow diagram article selection process.

Our synthesis results were derived from a total of 28 articles that are included in this review. Initially, 152 articles were identified by the full text review as potentially relevant and 19 articles were selected for extraction based on relevance to the IPT. Finally, nine additional publications were identified by scanning for key authors’ recent publications from external literature, with the aim of purposely searching for evidence that challenges or even disconfirms our emerging revised program theory. The primary inclusion criterion for all articles (including the additional nine articles) were that the articles exemplified high relevance to the initial program theory (whether supportive or challenging), and had sufficient detail regarding the process of participatory co-design to extract information on CMOCs. A flow diagram of the article selection process is detailed in Figure 2. All articles exemplified high relevance to the initial program theory, and had sufficient detail regarding the process of participatory co-design.

## Results

The theorizing has led us to findings from the rapid review that we believe are useful as a basis for elaborating some basic principles for AT design and implementation processes, and to make some recommendations to our stakeholders about how to move forward with the now urgent agenda for scale-up and spread, spurred by the burning platform of COVID-19. We discuss these findings under the two dimensions identified in our IPT. These two dimensions refer to the underlying motivations and the outcomes typically stated as aims for using PCD approaches that we found in the literature. For example, we found support for this distinction in the review by Fischer et al. (2020), where the “material” (acceptance, quality, adoption) and “soft” motivators (learning, understanding, feedback) related to our “epistemological dimension,” while the “normative motivators” (empowerment, social impact) related to our “ethico-political dimension.” Furthermore, Fischer et al.’s listed outcomes for PCD (Learning, Adjusted Design, Participation, and Adoption/Acceptance) also mapped on to our two dimensions.

### The Epistemological Dimension: Knowledge Integration

One of the most widely observed findings in the participatory co-design examples we reviewed in the literature, is that not only should “end-users” (i.e., older adults) be involved more in the research/design process, but that a wide array of “stakeholders” that are non-researchers/non-designers need to be involved as full participants as well (Shaw et al., 2017). As Donald et al. (2021) highlights,“[m]eaningful involvement in a co-design process can lead to intuitive and functional AT” (p. 11). Participatory co-design of technology supporting older adults aging in place requires ongoing evaluation with the involvement of older adults (Sumner et al., 2020). Furthermore, collaborative knowledge production or production of scientific knowledge can also be part of the successful co-design experience (Grigorovich et al., 2021). Exactly what mix of stakeholders (in addition to older adults themselves) should be involved is dependent on the specific context or setting of the AT implementation, that is, home, residential care or long-term care, but clearly informal care and support (e.g., family, friends, and neighbors) constitutes one common group. The underlying rationale for this pattern of stakeholder inclusion has two aspects: (i) a mutual orientation to knowledge; and (ii) a mutual orientation to action. The first aspect revolves around a mechanism we identify as *mutual awareness*. This mechanism operates by taking advantage of an ongoing, iterative, participatory/collaborative process of knowledge sharing (Context + or −) to integrate a variety of lived experiential perspectives, such that previously isolated perspectival orientations, for example, that of older adult users, informal carers, service professionals, AT designers, other academics, policy/decision-makers, are now articulated and become shared understandings (Procter et al., 2018). Thus we have:

**C-M-O-C** “If a genuine, participatory, collaborative research and development process is enacted (C), then the mechanism (M) of *mutual awareness* can effectively integrate diverse knowledge (O), leading to more successful and appropriate AT design and implementation”

This means that persons working and living from within each of the knowledge-to-action orientations, can come to use this newfound mutual awareness to more successfully orient future activities and contributions as the knowledge of what others perceive, understand, and do, becomes the context(s) for their own future action orientations (Greenhalgh, Jackson, et al., 2016; Greenhalgh, Shaw, et al., 2016). This mutual awareness, given the right contextual conditions (such as sustained engagement, regularized co-design activities, resources dedicated to inclusion and engagement processes, and a commitment by all project leaders to participation as a fundamental principle) should lead to more appropriate and effective technology solutions, as the end products embody and embed (integrate) knowledge of what will work for each of the main stakeholder groups. Connected to this, is the idea that various stakeholders act in the context of specific locations in a multi-level, complex adaptive system. The reciprocal nature of the aspects now becomes clear: while each stakeholder brings to the collaboration a certain combination of formal and informal knowledge that enriches the whole, through *mutual awareness*, each stakeholder takes away from the process new knowledge that they then must apply in their own action systems. Thus, older adults and informal carers take the knowledge they have gained and the AT produced and have to implement them in their own lived experiential context. Service providers must try to integrate their new understandings and support the technologies in the context of their everyday care processes and organizational work context. Technology developers have to learn to adapt their development processes, their production facilities, their marketing strategies, and their relations with regulatory bodies in light of the mutual awareness gained. Researchers have to adapt their methods, their dissemination practices and knowledge mobilization activities based on the new knowledge. Older adults must be considered as stakeholders with a high degree of expertise as well as the end-users, therefore they should be treated as experts and acknowledging their contributions to generate design and implementation insights; researchers, policy makers, and tech designers must be well trained on the use of language and behavioral skills to correctly promote engagement, collaboration, and cooperation via co-design approaches to achieve successful implementation co-design projects (Kramer et al., 2021).

Policy/decision-makers have to rethink their investment strategies, policy development, and regulatory functions, including technology assessment processes. This complex set of actions has to be coordinated and calibrated through a series of iterative cycles of *mutual learning*. Thus we have:

**C-M-O-C** “If iterative processes of knowledge-action-reflection cycles, involving all stakeholders are instituted and sustained (C), then the mechanism of *mutual learning* (M), can lead to more adaptable, flexible design processes that support ongoing implementation and enhance sustainability (O)”

What is important to note is that this collaborative sense-making integrates context-dependent practical knowledge (often largely tacit) with the type of representational-propositional knowledge often developed by researchers and technology developers to “map out” the domains relevant to the technology development and implementation process. A central figure in this iterative process is the role of “prototypes” and other artefacts of representation (e.g., office supplies, storyboards, logic maps) that provide a set of tools to engage collaboratively with the stakeholders in relation to the two (or more) types of knowledge (Wherton et al., 2012).

### The Ethico-Political Dimension

A common theme in the literature was the underlying aim of participatory co-design to *engage* older adults and their informal carers in order to respect their rights, autonomy and agency, and allow them to participate as fully as possible in guiding the design of the AT and their implementation. Sometimes the emphasis is on the somewhat nebulous concept of “empowerment” as a stand-alone outcome; at other times, the engagement process seems to be seen as the precondition (or “context” in realist terms) for successful knowledge integration (Auger et al., 2011). Furthermore, there are great advantages claimed for PCD methodologies beyond usability, such as its valuable contribution for “scalable services to be developed, deployed, tested, and evaluated with the end users, quickly and effectively prior to large scale implementation” (Ward et al., 2015, p. 14). While many PCD AT projects reference the original emphasis on “democratization” in the Scandinavian participatory design field, this is seldom worked into the core of the projects themselves.

This lack of a concrete democratization component in many studies is in itself an interesting finding, as there seems to be a drift away from the more explicit politics of participatory research and participatory design (Bygholm & Kanstrup, 2017), with more emphasis on the epistemological focus of action research. Grigorovich et al. (2021) point out that this contrast can be seen between approaches that take a participatory action research approach, as opposed to a “co-design” or “participatory design” approach. Some authors argue that, if we avoid stereotypes, while emphasizing empowerment and democratization, through mechanisms of learning, adjusted design, and the improvement of the sense of participation, these can lead to successful PCD experiences (Fischer et al., 2020).

Engagement is the overall strategy that is meant to: (1) “empower” social participation (Lopes et al., 2016); (2) address lack of sustainability (Zamir et al., 2018); (3) improve self-determination (Dupuy et al., 2016); (4) manage issues of consent, privacy, and human contact (Cahill et al., 2019); and, (5) respect the rights, dignity, and autonomy of older adults (Cahill et al., 2018). The question then becomes, what are the key mechanisms that underlie successful engagement of older adults in AT design and implementation? From our review of the literature and our data extractions and analyses, we can identify at least two important mechanisms at work. First, there is something about the “early and often” nature of concerted and well-planned iterative cycles of AT development that, in itself, leads to more successful engagement through a mechanism of *trust building*, that reinforces the message to older adult/informal carer stakeholders that their input is valued and is not merely a token gesture. It is clear that while the ideal of the iterative participatory process is to have stakeholders engaged at all stages of the process, exactly who is engaged, when they are engaged, and how they are engaged, varies widely, and is not often clearly stated in terms of the precise plan for implementing a consistent process of stakeholder engagement (Fischer et al., 2020; Grigorovich et al., 2021). Thus we have:

**C-M-O-C** “If consistent, repeated strategies of engagement are instituted in all stages of the development process (C), then the mechanism of *trust building* (M), can lead to active and effective participation of older adults in the co-design process (O)”

A major contextual influence on which older adults are engaged, when, and how, seems to be the perception of the researchers and AT developers concerning the capacity of the older adults to meaningfully participate in all aspects of the design process. Many AT projects underestimate the capacity of older adults to contribute to the design process; yet, it has been demonstrated that older adults are fully capable of doing so (Davidson & Jensen, 2013; Pradhan et al., 2020). Indeed, if older adults are actively engaged in participatory co-design projects, then it is more likely to ensure the usability, acceptability, and appropriateness of AT (LaMonica et al., 2021). This leads us to consider the second key mechanism identified in the literature we reviewed: *reciprocity*.

There is a clear connection between successful engagement of older adults and the enactment of a *reciprocal contribution* to the AT development process on behalf of older adult participants. Meaningful engagement seems to happen when older adults themselves feel they are contributing positively to the development of the AT. This goes beyond the typical contribution of “information” in the form of answers to interview questions or survey items; it denotes actual changes to the AT development, sometimes referred to as “adjusted design” (Fischer et al., 2020) that are driven directly by the desires and ideas of the older adults themselves. A meaningful social inclusion approach offers real opportunities of reciprocity (Span et al., 2018) and effective knowledge integration through participative engagement (Scandurra & Sjölinder, 2013). This operates as a kind of proof of the value of their contributions, as materialized in adjusted design (Fischer et al., 2020). It also validates their self-efficacy and self-worth as members of society that are actively co-producing technological solutions that will support real needs (Kort et al., 2019; Marston et al., 2020), enhancing their own quality of life. Thus we have:

**C-M-O-C** “If AT researchers and developers avoid ageist stereotyping and assume older adults are capable as co-designers (C), then the mechanism of *reciprocal contribution* (M), can lead to more older adults feeling empowered and self-efficacious, as they see their contributions reflected in adjusted design and mutual learning (O)”.

## Discussion

This early work in our project has led us to several conclusions, both in terms of some important messages we think are already apparent for key stakeholders attempting to implement ATs for older adults more widely and more sustainably, and in terms of the future directions of our research. First, there is now abundant evidence, from the academic literature, that AT need to be developed *with* older adults and their caregivers as part of a participatory co-design process, if ATs for older adults are to be more effective, usable, accepted, and thus adopted, and not abandoned (Wherton, Sugarhood, Procter, Greenhalgh, 2015; Wherton, Sugarhood, Procter, Hinder, et al., 2015). Second, older adults, despite the prejudices and concerns regarding their capacity to engage in participatory co-design processes, are not only well able to do so, but have the capacity to be key partners in the design process (Peine & Neven, 2019). Third, context is paramount: a central rationale for participatory co-design is that it allows the design process to account for the context-sensitive nature of the settings within which, and with whom, specific AT are meant to be integrated. A context with robust ethical governance, and deep commitment to participatory principles, is essential to ensure effective knowledge integration. (Grigorovich et al., 2021; Martin et al., 2013). Fourth, the systems that form the overall context within which AT implementation processes must be initiated, are complex, multi-level adaptive systems, that have recursive feedback and causal loops. An implication of this last point is that AT implementation requires key stakeholders at all levels of this multi-level system to be integral participants in an overall co-design process. In fact, we argue here that this latter point may be one of the missing links in the literature. As Fischer et al., point out, in relation to the outcomes of acceptability and adoption, PCD methodologies have a mixed record. It may be that the lack of a multi-stakeholder approach to PCD is what is holding back some of these initiatives, as PCD focuses exclusively on older adults, and not on their local residential context (including social support networks), their formal care systems, and the regulatory environment that is implicated in the sustainability of AT adoption and implementation. For older adults, specifically, a robust and genuine PCD approach that becomes the standard for AT design can mean that they will have many more opportunities to shape their own technological futures, leading to lives that are more independent, staying at home longer, and having enhanced opportunities for empowering experiences with technology.

## Strengths and Limitations

Although we are confident that we have surveyed many of the key articles relevant to our program theory, ironically, relying too heavily on systematic search procedures can lead to the inadvertent exclusion of relevant literature. While some of our additional nine articles were simply added due to their publication date being later than our original search parameters, we also identified articles that were not in our initial set of abstracts identified, due to several factors that are related to the strictures of systematic searches based on key words and electronic databases. With a topic that is theoretically driven, such as our one, it is more than likely that we have overlooked key articles, and even whole sub-fields of research (e.g., we suspect that some of the approaches to co-design rooted more deeply in arts and humanities-based approaches may have been missed) simply aren’t found because they either aren’t included in the databases we chose, or the language used to describe their work in titles and abstracts escaped our keyword parameters. To mitigate this limitation, we decided to use several additional search strategies, including: snowball citation, key journal searches, and expert recommendations. In fact, one of our expert panelists identified an emerging group of authors in a field they refer to as “socio-gerontechnology,” many of whose work was not picked up in our systematic search of databases. As our intention was to deepen the theoretical understanding of participatory co-design of AT with older adults, these sources of knowledge were added external to our original search parameters. While this may be seen as itself a limitation (a bending of the rules of inclusion), we feel that it significantly enhanced our final product. Another limitation of our study, and consistent with other recent reviews (Fischer et al., 2020; Grigorovich et al., 2021; Merkel & Kucharsky, 2019), is that we still have a paucity of concrete descriptions from PCD study reports about the details of their engagement processes, including barriers and challenges faced. One significant issue is that while we know that older adults constitute a very diverse group, marked by multiple and intersecting identities, very little information is available about how these different sub-populations of older adults engage or do not engage with PCD processes. This is a significant contextual factor that needs to be taken into account when evaluating the effectivity of any specific engagement strategy in PCD work.

The main strength of our review has been the theoretical perspective of the realist approach, which has allowed us to isolate some of the key underlying mechanisms, which seem to contribute to successful PCD approaches, given the right contextual circumstances. Future research should make substantial efforts to evaluate the PCD processes used in relation to these mechanisms, and to collect relevant contextual data that helps to explain diverse patterns of outcomes.

## Conclusion

This leads to the ultimate lesson we are beginning to see as framing our next steps in future research: that “ATs” are not discrete material objects, but rather that they are complex actants in a distributed socio-technical network (Peine et al., 2021). This means that PCD doesn’t start and end when an AT “product” is completed, but instead follows the entire process of implementation in those complex, adaptive socio-technical networks, as the AT are adopted, scaled-up, spread and sustained. At each level in these systems, we require cognisant, reflective agents to collaborate and negotiate the varied, uncertain, and challenging contexts of implementation. An overall collaborative action research approach is regarded as the most viable and appropriate framework for carrying out this type of engaged, iterative cycle of learning and action, to realize the full potential of AT for improving the health, well-being, and quality of life for older adults and the sustainability of health and social care systems in the future.
